# Supplementary material for: Machine-learned epidemiology: real-time detection of foodborne illness at scale
Source: NPJ Digit Med. 2018 Nov 6;1:36. doi: 10.1038/s41746-018-0045-1 (PMC6550174; doi:10.1038/s41746-018-0045-1)
Supplement: Supplementary file 1 — Supplemental Material [file 41746_2018_45_MOESM1_ESM.pdf]

Supplementary Methods

Restaurant inspections

The two cities where we ran the experiment use different grading systems for restaurant inspections. In Chicago, restaurants are either given a Pass, Pass with Conditions (PC) or Fail. Pass is essentially a clean bill of health. Failures occur when serious or critical violations are found that cannot be fixed during the inspection. PC is given when serious or critical violations are found that are fixed during the inspection. Therefore, we group PC and Fail together into *unsafe* category, as they both involve significant health code violations.

In Las Vegas, possible inspection grades are A, B, C, or Closed. Restaurants that don't receive an A are deemed to pose serious threats to public health and are immediately scheduled for re-inspection to determine if corrective actions have been taken. Therefore, we considered grades B, C, and Closed to be *unsafe* outcomes. We grouped all the restaurants graded A/Pass together into the Safe set, and grouped the restaurants graded B, C/PC, or Fail/Closed into the Unsafe set. These safe/unsafe labels were used to assess the accuracy of FINDER predictions.

Some of the restaurant inspections were purely administrative in nature, e.g., those conducted as part of starting new businesses before they are granted a license. Naturally, we excluded the results of such inspections from analysis throughout the paper.

Inspection Methodology of the Southern Nevada Health Department (SNHD), Las Vegas, Nevada

In Las Vegas, restaurants are routinely inspected once a year and can also be inspected as a result of a complaint, but this is rare (Las Vegas received 15 complaints during the 4-month experimental period). Inspectors are given a list of restaurants to inspect at the beginning of the year and must complete all inspections by the end of the year, in whatever order they choose. A routine inspection is a risk-based process addressing the food establishment's control over the following five areas of risk for foodborne illness: personal hygiene, approved food source, proper cooking temperatures, proper holding times and temperatures, and sources of contamination. Violations are weighted based on their likelihood to directly cause a foodborne illness, and are divided into critical violations (at 5 demerits each, e.g., food handlers not washing hands between handling raw food and ready to eat food), major violations (at 3 demerits each, such as hand sink not stocked with soap), and good food management practices (no demerit value, e.g., leak in the hand sink). Demerits are converted to letter grades, where 0-10 is an A, 11-20 is a B, 21-39 is a C, and 40+ is an F (immediate closure). A repeated violation of a critical or major item causes the letter grade to drop to the next lower rank. Any grade less than an A is required to undergo a re-inspection to confirm all critical and major violations have been corrected.

Whenever a food establishment was identified by FINDER, the assigned inspector was instructed to conduct a standard routine inspection on two restaurants: the FINDER-flagged restaurant and a matched restaurant from the routine inspection list. Matched restaurants were selected at random based on their location and permit type to match the FINDER-flagged restaurants. The inspectors were not aware of which restaurant was flagged by FINDER, so that each facility received the same risk-based inspection. The venue owner/manager was also not aware of the

experiment and was told by the inspector that a routine inspection was being conducted. Google staff was not privy to inspector assignment. After the end of the experiment, the SNHD staff collected information about the number and the type of violations found at the FINDER-identified restaurants and at the matched restaurants. Only this set of restaurants had detailed information about the count and severity of violations that could be compared across the two cities, and thus only these restaurants were used in the analysis of violation counts. Matched restaurants were included in the ROUTINE subset of the BASELINE group.

Inspection Methodology of the Chicago Department of Public Health (CDPH), Chicago, Illinois

In Chicago, a restaurant inspection can result in one of three outcomes: Pass, Pass with Conditions (PC), and Fail. A venue passes the inspection when there are no serious or critical violations found, passes with conditions when serious or critical violations are found that are corrected during the inspection, and fails the inspection when serious or critical violations are found but are not corrected on the spot. A failed inspection results in immediate closure of the establishment and suspension of the establishment's license.

CDPH performs initial health inspections prior to the opening of a food establishment, and assigns a risk level, either 1, 2 or 3, to the establishment that will determine the frequency of future routine inspections. Establishments at risk level 1 (the highest risk) are inspected twice a year, those at level 2 are inspected once a year, and those at level 3 are inspected every other year. During an inspection, inspectors look for serious or critical violations. A serious violation indicates a "potential health hazard" that must be corrected within a timeline established by the inspector, and if it has not been remedied on re-inspection, the establishment is closed. A critical

violation poses “an immediate health hazard” and must be fixed while the inspector is present or else the restaurant is closed. For a complete list of violations, see Supplementary Table S1. For the violation count analyses, critical violations were grouped with critical violations in Las Vegas, and serious violations were grouped with major violations in Las Vegas.

In addition to these routine inspections, restaurants can also be inspected when CDPH receives a complaint. Chicago has an advanced complaint system that includes complaints generated from phone calls, Foodborne Chicago (a social media mining system), as well as a predictive analytics system. Complaints produced by any of these mechanisms receive higher inspection priority than routine inspections.

All FINDER-identified restaurants were inspected according to the standard protocol. The inspectors were not aware of the experiment, and therefore were not aware which restaurants were flagged by FINDER, so that each establishment received the same risk-based inspection. The venue owner/manager was also not aware of the experiment and was told by the inspector a routine inspection was being conducted. Google staff was not privy to inspector assignment.

Sensitivity Analyses

We conducted a series of sensitivity analyses to assess the robustness of our method. First, we analyzed the results for each of the cities separately, and observed similar results; FINDER restaurants were more likely to be unsafe than BASELINE restaurants (Supplementary Table S3).

In Chicago, if an issue is found during an inspection, a re-inspection is conducted shortly thereafter to determine whether the establishment has complied with the requests of the inspectors. Since this type of inspection is slightly different from a typical routine or complaint-based inspection, we conducted a sensitivity analysis where these re-inspections were excluded. The results were qualitatively similar, except the increased sensitivity of FINDER was no longer statistically significant when compared to complaints (Supplementary Table S4).

In our main experiment, we considered restaurants as unsafe if they either passed an inspection with conditions or failed outright. We also assessed FINDER's precision in identifying unsafe restaurants under a more restrictive definition of unsafe, namely, only considering restaurants with the most serious violations (grade C or worse in Las Vegas or a Closure in Chicago) as unsafe. In this analysis, FINDER again identified a higher fraction of unsafe restaurants than routine and complaint-based inspections (Supplementary Tables S5-S7).

Additionally, we compared the adjusted mean number of critical and major violations within each city and found similar results (Supplementary Tables S8 and S9). Finally, we used a multinomial logistic regression with three possible values of the dependent variable: safe (grade A in Las Vegas or Pass in Chicago), unsafe (grade C or worse in Las Vegas or Fail in Chicago), and a new semi-safe category (grade B in Las Vegas or Pass with Conditions in Chicago). In this analysis, we again found that both unsafe and semi-safe outcomes were more likely to occur in FINDER-flagged restaurants than in BASELINE restaurants (Supplementary Table S10).

Additionally, we conducted a sensitivity analysis where we compared the likelihood of being deemed unsafe among FINDER restaurants versus all restaurants, including both FINDER and BASELINE restaurants. Here we find that again, FINDER-identified restaurants are more likely to be closed than FINDER+BASELINE restaurants. Finally, we compared FINDER+BASELINE vs BASELINE to examine the additive effect of including FINDER in routine inspections. Here, the direction of the results are the same, where adding FINDER into the baseline inspections increased the rate of unsafe restaurants that were identified, however, given the relatively small number of FINDER restaurants, the impact is also relatively small (Supplementary Table S11).

Supplementary Tables

Table S1. List of all critical and serious violations that could arise during an inspection in Chicago Department of Public Health.

Violation Number	Health Standard to Be Met	Violation Type
1	· All food shall be from sources by health authorities and safe for human consumption.<br>· Shellfish shall be obtained from an approved source and kept in their original package until sold.<br>· Molluscan shell stock shall be obtained in containers bearing legible source identification tags or labels.	Critical
2	· All food establishments that prepare, sell, or store hot food shall have adequate hot food storage facilities.<br>· All food establishments that display, prepare, or store potentially hazardous food shall have adequate refrigerated food storage facilities.	Critical
3	· All hot food shall be stored at a temperature of 140°F or higher.<br>· All cold food shall be stored at a temperature of 40°F or less.	Critical
4	All food shall be protected from contamination and the elements, and so shall all food equipment, containers, utensils, food contact surfaces and devices, and vehicles	Critical
5	No person affected with or carrying any disease in a communicable form or afflicted with boils, infected wounds, sores, acute respiratory infection, or intestinal disorder shall work in any area of a food establishment in any capacity where there is a likelihood of that person contaminating food or food contact surfaces.	Critical
6	All employees who handle food shall wash their hands as often as necessary to maintain a high degree of personal cleanliness and should conform to hygienic practices prescribed by the Board of Health.	Critical
7	Hand washing of all tableware and drinking utensils shall be accomplished by the use of warm water at a temperature of 110°F to 120°F containing an adequate amount of detergent effective to remove grease and solids.	Critical
8	Equipment and utensils should get proper exposure to the sanitizing solution during the rinse cycle. Bactericidal treatment shall consist of exposure of all dish and utensil surfaces to a rinse of clean water at a temperature of not less than 180°F.	Critical
9	All food establishments shall be provided with an adequate supply of hot and cold water under pressure properly connected to the city water supply.	Critical
10	In food establishments, there shall be adequate sewage and waste water disposal facilities that comply with all requirements of the plumbing section of the Municipal Code of Chicago.	Critical
11	Adequate and convenient toilet facilities shall be provided. They should be properly designed, maintained, and accessible to employees at all times.	Critical
12	Adequate and convenient hand washing facilities shall be provided for all employees.	Critical
13	All necessary control measure shall be used to effectively minimize or eliminate the presence of rodents, roaches, and other vermin/insect infestations.	Critical
14	A separate and distinct offense shall be deemed to have been committed for each Serious violation that is not corrected upon re-inspection by the health authority.	Critical
**Violation Number**	**Violation Short Description**	**Violation Type**
15	Food once served to a consumer shall not be re-served, with the exception of packaged food remaining in its original, unopened package.	Serious
16	All food should be properly protected from contamination during storage, preparation, display, service, and transportation.	Serious

17	Thawing frozen food for further processing shall be accomplished by storage in a refrigerator at 40°F or less, or by other approved method.	Serious
18	All necessary control measures shall be used to effectively minimize or eliminate the presence of rodents, roaches, and other vermin and insects on the premises of all food establishments, in food-transporting vehicles, and in vending machines.	Serious
19	The area outside of the establishment used for the storage of garbage shall be clean at all times and shall not constitute a nuisance.	Serious
20	All garbage and rubbish containing food wastes shall, prior to disposal, be stored in metal containers with tight fitting lids and shall be kept covered except when opened for the disposal or removal of garbage.	Serious
21	A certified food service manager must be present in all establishments at which potentially hazardous food is prepared or served.	Serious
22	All dishwashing machines shall maintain proper water pressure and must be provided with suitable thermometers, chemical test kits, and gauge cocks.	Serious
23	Dishes and other utensils shall be rinsed or scraped to remove gross food particles and other soil before washing.	Serious
24	All dishwashing machines must be of a type that complies with all requirements of the plumbing section of the Municipal Code of Chicago and Rules and Regulation of the Board of Health	Serious
25	Only such poisonous and toxic materials as are required to maintain sanitary conditions may be used in food establishments and they shall not be used in any hazardous manner.	Serious
26	When toilet and lavatory facilities are provided for the patrons of food establishments, such facilities shall be adequate in number, convenient, accessible, properly designed, and installed according to the municipal code.	Serious
27	In all food establishments, toilet facilities shall be kept clean and in good repair and shall include an adequate supply of hot and cold or tempered water, soap, and approved sanitary towels or other approved hand-drying devices.	Serious
28	One copy of the Food Inspection Report Summary must be displayed and visible to all customers.	Serious
29	A separate and distinct offense shall be deemed to have been committed for each Minor violation that is not corrected upon re-inspection by the health authority.	Serious
30	Food not in original container, not properly labeled; no customer advisory posted as required	Minor
31	Clean multi-use utensil and single service articles improperly stored; re-use of single service articles	Minor
32	Food and non-food contact surfaces improperly designed, constructed and maintained	Minor
33	Food and non-food contact equipment/utensil not clean and free of abrasive detergents	Minor
34	Floor: poorly constructed, not drained, not clean, not in good repair, covering improperly installed, no dustless cleaning methods	Minor
35	Walls, ceilings, attached equipment: poorly constructed, not in good repair, unclean surfaces, no dustless cleaning methods	Minor
36	Lighting: not provided as required, fixtures not shielded	Minor
37	Dressing rooms: no lockers provided, located and used; no separation from living/sleeping quarters	Minor
38	Ventilation: rooms and equipment not vented as required; Plumbing: not installed and maintained.	Minor
39	Clean, soiled linen not properly stored	Minor
40	Refrigeration thermometers not provided or conspicuous	Minor
41	Premises not maintained free of litter and /or unnecessary articles cleaning equipment improperly stored	Minor
42	Appropriate method of handling of food (ice); not minimized no hair restraints and clean apparel not worn	Minor
43	Food (ice) dispensing utensils not properly stored when in use	Minor
44	Unauthorized persons in food preparation area	Minor
45	Food handler requirements not met	Minor

Table S2. List of all critical and major violations that could arise during an inspection by Southern Nevada Health District.

Violation	Health Standard to Be Met	Violation Type
1	Verifiable time as a control approved procedure when in use. Operational plan, waiver, or variance approved and followed when required. Operating within the parameters of the health permit.	Critical
2	Handwashing (as required, when required, proper glove use, no bare hand contact of ready to eat foods). Foodhandler health restrictions as required.	Critical
3	Commercially manufactured food from approved source with required labels. Parasite destruction as required. Potentially hazardous foods/time temperature control for safety foods (PHF/TCS) received at proper temperature.	Critical
4	Hot and cold running water from approved source as required.	Critical
5	Imminently dangerous cross connection or backflow. Wastewater and sewage disposed into public sewer or approved facility.	Critical
6	Food wholesome, not spoiled, contaminated or adulterated.	Critical
7	PHF/TCSs cooked and reheated to proper temperatures.	Critical
8	PHF/TCSs properly cooled.	Critical
9	PHF/TCSs at proper temperature during storage, display, service, transport, and holding.	Critical
10	Food and warewashing equipment approved, properly designed, constructed and installed.	Major
11	Food protected from potential contamination during storage and preparation.	Major
12	Food protected from potential contamination by chemicals. Toxic items properly labeled, stored, and used.	Major
13	Food protected from potential contamination by employees and consumers.	Major
14	Kitchenware and food contact surfaces of equipment properly washed, rinsed, sanitized, and air dried. Equipment for ware washing operated and maintained. Sanitizer solution provided and maintained as required.	Major
15	Handwashing facilities adequate in number, stocked, accessible, and limited to handwashing only.	Major
16	Effective pest control measures. Animals restricted as required.	Major
17	Hot and cold holding equipment present, properly designed, maintained and operated.	Major
18	Accurate thermometers (stem & hot/cold holding) provided and used.	Major
19	PHF/TCSs properly thawed. Fruits and vegetables washed prior to preparation or service.	Major
20	Single use items not reused or misused.	Major
21	Person in charge available and knowledgeable/management certification. Food handler card as required. Facility has an effective employee health policy.	Major
22	Backflow prevention devices and methods in place and maintained.	Major
23	Grade card and required signs posted conspicuously. Consumer advisory as required. Records/logs maintained and available when required. Nevada Clean Indoor Air Act compliant. PHF/TCSs labeled and dated as required. Food sold for offsite consumption labeled properly.	Major

Table S3. Ability of FINDER to detect unsafe restaurants as compared to BASELINE rate, stratified by City

	**FINDER n=71**	**BASELINE n=5,809**	**OR [95% CI]**	**p-value**
**Chicago**	37 (52.1%)	1,971 (33.9%)	2.11 [1.32-3.37]	0.002
	**FINDER n=61**	**BASELINE n=4,977**	**OR [95% CI]**	**p-value**
**Las Vegas**	32 (52.5%)	691 (13.9%)	4.20 [2.46-7.17]	<0.001

Table S4. Ability of FINDER to detect unsafe restaurants as compared to BASELINE, COMPLAINT and ROUTINE inspections when excluding re-inspection results. *The complaint comparison involved only restaurants in Chicago, therefore for FINDER, n=71.

	FINDER number unsafe (%)	Comparison group number unsafe (%)	Odds Ratio [95% CI]	p-value
**BASELINE n=9,501**	69 (52.3%)	2,480(26.1%)	2.67 [1.87-3.83]	<0.001
**COMPLAINT* n=994**	37 (52.1%)	464 (46.7%)	1.24 [0.77-2.01]	0.38
**ROUTINE n=8,507**	69 (52.3%)	2,016 (23.7%)	2.80 [1.96-4.00]	<0.001

Table S5. Ability of FINDER to detect unsafe restaurants with more restrictive definition of unsafe (Fail in Chicago, C or Failure in Las Vegas) as compared to all other inspections

	**FINDER n=132**	**BASELINE n=10,786**	**Odds Ratio [95% CI]**	**P-value**
**Overall, number unsafe (%)**	34 (25.8%)	1,384 (12.5%)	2.53 [1.66-3.86]	<0.001
**Restaurant Risk Level**				
**High, number unsafe (%)**	22 (26.2%)	1,080 (18.9%)	1.81 [1.10-2.98]	0.02
**Medium, number unsafe (%)**	10 (25.6%)	213 (9.2%)	4.23 [1.90-9.40]	<0.001
**Low, number unsafe (%)**	2 (22.2%)	55 (2.0%)	8.18 [0.94-71.18]	0.06

Table S6. Ability of FINDER to detect unsafe restaurants with more restrictive definition of unsafe (Fail only) as compared to complaint-based inspections in Chicago

	**FINDER n=71**	**COMPLAINTS n=1,291**	**Odds Ratio [95% CI]**	**P value**
**Overall, number unsafe (%)**	22 (31.0%)	288 (22.3%)	1.57 [0.93-2.65]	0.09
**Restaurant Risk Level**				
**High, number unsafe (%)**	16 (28.1%)	211 (22.2%)	1.36 [0.75-2.48]	0.31
**Medium, number unsafe (%)**	5 (41.7%)	60 (20.5%)	2.77 [0.85-9.05]	0.09
**Low, number unsafe (%)**	1 (50.0%)	17 (34.7%)	1.88 [0.11-32.0]	0.66

Table S7. Ability of FINDER to detect unsafe restaurants with more restrictive definition of unsafe (Fail only in Chicago and C/Failure in Las Vegas) as compared to ROUTINE inspections

	FINDER n=132	ROUTINE n=10,463	Odds Ratio [95% CI]	P value
**Overall, number unsafe (%)**	34 (25.8%)	1,060 (11.2%)	2.56 [1.68-3.89]	<0.001
**Restaurant Risk Level**				
**High, number unsafe (%)**	22 (26.2%)	886 (18.3%)	1.84 [1.12-3.05]	0.02
**Medium, number unsafe (%)**	10 (25.6%)	154 (7.6%)	4.22 [1.89-9.41]	<0.001
**Low, number unsafe (%)**	2 (22.2%)	38 (1.4%)	9.51 [1.21-74.64]	0.03

Table S8. Las Vegas Violation Counts. *Adjusted mean violation count, accounting for Restaurant Risk Level.

	**FINDER*** **n=61**	**BASELINE*** **n=39**	**P-value**
**Critical Violation Count**	1.29	0.87	0.15
**Major Violation Count**	4.10	2.94	0.08

Table S9. Chicago Violation Counts. *Adjusted mean violation count, accounting for Restaurant Risk Level.

	**FINDER*** **n=71**	**BASELINE*** **n=5,809**	**p-value**
**Critical Violation Count**	0.31	0.20	0.07
**Major Violation Count**	0.39	0.52	0.14

Table S10. Ability of FINDER to detect restaurants that receive a pass with conditions or failing grade, using multinomial logistic regression with City and Restaurant Risk Level fixed effects as compared to BASELINE inspections. *Relative Risk Ratio

	Pass	Pass with Conditions	Fail
**FINDER**<br>**[RRR* (95% CI)]**	63 (47.7%)<br>-	35 (26.5%)<br>[2.84 (1.86-4.34)]	34 (25.8%)<br>[3.32 (2.12-5.18)]
**BASELINE**<br>**[reference]**	8,124 (75.3%)	1,314 (12.2%)	1,348 (12.5%)

Table S11. Comparison of identification of unsafe restaurants for FINDER vs FINDER+BASELINE and BASELINE vs FINDER+BASELINE.

	**FINDER+ BASELINE N=10,918**	**FINDER N=132**	**OR (95% CI)**	**BASELINE N=10,786**	**OR**
**Overall, number unsafe (%)**	2,731 (25.0)	69 (52.3)	2.99 [2.10-4.25]	2,662 (24.7)	1.02 [0.95-1.08]
**Risk Level**					
**High**	1,951 (33.7)	42 (50.0)	1.94 [1.26-3.00]	1,909 (33.5)	1.01 [0.94-1.09]
**Medium**	559 (23.7)	23 (59.0)	5.29 [2.72-10.28]	536 (23.1)	1.03 [0.90-1.19]
**Low**	221 (8.0)	4 (44.4)	7.24 [1.77-29.70]	217 (7.9)	1.01 [0.83-1.24]
